# Oral application of magnesium‐L‐threonate enhances analgesia and reduces the dosage of opioids needed in advanced cancer patients—A randomized, double‐blind, placebo‐controlled trial

**DOI:** 10.1002/cam4.4922

**Published:** 2023-01-26

**Authors:** Siyin Wu, Tian Jin, Bingjie Ma, Yun Ji, Xuehua Huang, Peiliang Wang, Xiaoming Liu, Boris V. Krylov, Xianguo Liu, Ke Ma

**Affiliations:** ^1^ Department of Pain Medicine Xinhua Hospital affiliated to Shanghai Jiaotong University, School of Medicine Shanghai China; ^2^ Pavlov Institute of Physiology of the Russian Academy of Sciences Saint Petersburg Russia; ^3^ Department of Physiology and Pain Research Center, Zhongshan School of Medicine Sun Yat‐sen University Guangzhou China; ^4^ Guangdong Province Key Laboratory of Brain Function and Disease Guangzhou China

**Keywords:** cancer pain, constipation, magnesium‐L‐Threonate, morphine, analgesic

## Abstract

**Purpose:**

To investigate the effects of oral administration of magnesium‐L‐threonate, a novel magnesium compound, on the analgesic effect of opioids in patients with advanced cancer.

**Methods:**

We performed a prospective, randomized, double‐blind trial at a tertiary hospital in Shanghai, China. Eligible cancer patients who took opioids orally were assigned randomly to receive L‐TAMS capsules (1.5 g or 2.0 g according to weight) or a placebo (starch capsules). The primary outcome was the increase in the daily oral dose of morphine in each of the two groups, measured at 7, 14, 21, 30, 60, and 90 days during this trial.

**Results:**

A total of 116 patients from the oncology and pain departments, including inpatients and outpatients, were screened; 83 were enrolled. The increases in daily morphine doses began to differ from day 30 (L‐TAMS group 9.85 mg/d vs. Placebo group 20.49 mg/d, *p* < 0.05); the differences persisted on day 60 (L‐TAMS group 15.96 mg/d vs. Placebo group 29.06 mg/d, *p* < 0.05) and on day 90 (L‐TAMS group 21.20 mg/d vs. Placebo group 40.44 mg/d, *p* < 0.01).

**Conclusions:**

L‐TAMS outperforms a placebo in enhancing the analgesic effect of opioids and reducing the necessary opioid dosage. Moreover, L‐TAMS can significantly relieve opioid‐induced constipation. These advantages may be beneficial to patients with advanced cancer.

## Graphical abstract

A randomized, double‐blind placebo‐controlled prospective clinical trial. Oral magnesium‐L‐threonate enhances the analgesia and reduces the dosage of opioids needed in advanced cancer patients. Magnesium‐L‐threonate inhibits or eases morphine tolerance. Magnesium‐L‐threonate relieves opioid‐induced constipation

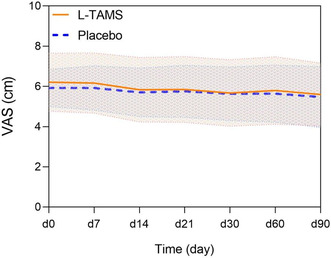

## 1 BACKGROUND

With modern advances in cancer therapy, the mean survival of cancer patients is increasingly prolonged, and many individuals are living with cancer pain as a result. According to statistics from the WHO, at least 5.5 million cancer patients worldwide suffer from pain every day, and 70%–90% of patients with advanced cancer are experiencing or have experienced pain; in 30%–45% of these cases, the pain is severe.^1^ Opioids are recommended in various guidelines as the preferred treatment for cancer pain. However, long‐term usage of opioids may contribute to opioid tolerance, which weakens the analgesic effect and increases the risk of adverse events. Opioid‐induced constipation (OIC) is the most common gastrointestinal reaction caused by opioids, affecting 23%–63% of users; patients develop only weak tolerance or no tolerance to this side effect.^2^, ^3^

Although the National Comprehensive Cancer Network (NCCN) guidelines recommend adding adjuvant analgesics (e.g., gabapentin, pregabalin, or corticosteroids) if opioids are insufficient for analgesia, repeated usage of these drugs may bring adverse reactions. Additionally, even though these combination drugs have shown benefits in pain control, some patients with intermediate or advanced cancer still receive no significant clinical advantage from these drugs in terms of decreasing opioid dosage and opioid‐related adverse events.^1^, ^2^, ^4^, ^5^

Animal studies have shown that abdominal or intravenous injection of magnesium sulfate (MgSO_4_) increases the effectiveness of opioids against chronic and acute pain and inhibits opioid dependence and tolerance.^6^, ^7^, ^8^ In recent years, a new magnesium compound known as magnesium‐L‐threonate (L‐TAMS) has been developed. Some studies have confirmed that L‐TAMS can effectively counteract neuroinflammation, normalize synaptic potentiation, correct magnesium ion (Mg^2+^) deficiency, and prevent chemotherapy‐induced neuropathic pain by increasing intracellular magnesium ions and inhibiting the tumor necrosis factor‐α/nuclear factor‐κB pathway^9^, ^10^, ^11^; mechanistically, the neuroinflammatory response plays a role in the development of neuropathic cancer pain and opioid tolerance. On this basis, we conducted this double‐blind, placebo‐controlled randomized clinical trial in China to explore the effect of L‐TAMS on the analgesic effect of oral opioids in patients with advanced cancer.

## 2 METHODS

### 2.1 Study design and participants

Our trial was a randomized, double‐blind, placebo‐controlled, longitudinal superiority trial performed at one tertiary hospital in Shanghai, China (Xinhua Hospital, affiliated with Shanghai Jiaotong University School of Medicine). Eligible participants were 18–80 years old^12^ and had a 24 h average Visual Analogue Scale (VAS) score (0–10) ≥ 4 or breakthrough cancer pain (BTcP) episodes ≥3 times per day. BTcP was defined according to the simple clinical diagnostic algorithm proposed for this purpose by Davies et al.^13^ Subjects included cancer patients who were taking morphine sulfate sustained‐release tablets or other opioids orally (oral dose: 20–200 mg morphine equivalents/24 h); those who took opioids by intravenous infusion, transdermal patches, or implantable drug delivery systems (IDDS) were excluded. All drugs were converted to morphine equivalent doses according to the dose conversion table of the NCCN Clinical Practice Guidelines in Oncology—Adult Cancer Pain (Version 3.2019)^5^ and voluntarily signed informed consent for this trial. Furthermore, adequate renal and hepatic function, satisfactory medication and visit compliance, the ability to communicate objectively and clearly, and a life expectancy of at least 3 months were required. More details can be found in Data S1.

Patients who were allergic to L‐TAMS or could not tolerate normal oral doses (suffering side effects such as nausea, vomiting, dizziness, palpitations or other obvious discomfort) were excluded; patients who were predisposed to allergies were also excluded, as were those with past or present drug abuse or drug addiction. Other exclusion criteria included women who were breastfeeding or pregnant, patients (men included) who were trying to conceive within 1 month after this trial, patients who had participated in any drug trial (including trials of L‐TAMS) within 3 months prior to this trial, and those who had significant traumatic pain or postoperative incision pain in addition to cancer pain.

### 2.2 Randomization and masking

Eligible participants (*n* = 83) were randomly assigned (1:1) to receive either L‐TAMS or a corresponding placebo (capsules containing food starch); the assignments were based on a computer‐generated block randomization list, which was prepared by an individual not involved in this trial. A company independent of the trial prepared the drugs based on a random list of numbers, with each drug package having its unique identification number. The researchers, who were not involved in preparing these packages, distributed them to the patients. All patients, clinicians, and research staff who collected or analyzed the data were blinded to treatment assignments until the database is locked. In a severe adverse event requiring immediate identification of the specific drug used, emergency unblinding was permitted. Once a patient's treatment was unmasked, the subject was removed from the trial and classified as a dropout; the outcome was reported to the clinical supervisor.

### 2.3 Procedures

All participants were already taking morphine orally when the trial began. In addition to their existing regimens, the randomly assigned experimental group took an L‐TAMS preparation (Wuxi Brain Magnesia Biomedical Technology Co, Ltd.) orally, at a dose of 1.5 g (weight < 70 kg: red capsule) or 2.0 g (weight > 70 kg: blue capsule)^9^; the placebo group received a uniform oral dose of 1.0 g (weight < 70 kg: red capsule; weight > 70 kg: blue capsule) starch preparation. All the abovementioned capsules were identical in appearance except for the color. The capsules were to be taken once daily (30 min before the first morphine administration) for 12 weeks or until patients voluntarily withdrew from the trial, had unexpected or serious toxic or side effects, became pregnant, died, or were required by the investigator to withdraw from the trial for safety reasons. Patients were allowed to adjust their oral morphine dosage reasonably to their pain levels during this study (each adjustment needed to be approved and documented by their attending physicians). In principle, neither group took any additional opioids, nonsteroidal anti‐inflammatory drugs or other adjuvant analgesics. However, when patients had a VAS score > 7, oral Oxycodone, and Acetaminophen Tablets (Mallinckrodt Inc Co, Ltd.) was used in a standardized manner to control BTcP; the dose of tablets was selected according to the dose of oral morphine.

The primary outcome of this trial was the increase in daily oral morphine use. Considering that patients could voluntarily adjust their morphine doses according to pain intensity, the VAS score for average pain was not used as the main index to evaluate the efficacy of L‐TAMS; it was used only for screening participants, evaluating patients' response to dosage changes, and guiding rational drug use under some special circumstances. Other efficacy measures included the frequency and severity of BTcP.

The Patient Health Questionnaire‐9 (PHQ‐9)^14^ and Generalized Anxiety Disorder‐7 (GAD‐7)^15^ were used to assess patients' depressive and anxious symptoms, respectively. After being instructed by researchers in the use of the scales, patients independently used the PHQ‐9 and GAD‐7 for self‐evaluation at baseline and 7, 14, 21, 30, 60, and 90 days after the beginning of this trial, and researchers accurately recorded each score.

Opioid‐induced constipation (OIC) was one of the secondary outcomes; the Wexner constipation score^16^ was used to numerically measure constipation at baseline and at 7, 14, 21, 30, 60 and 90 days during this trial. Patients summarized their symptoms in the past 7 days, including the number and timing of defecation, the degree of difficulty in defecation, the frequency of incomplete evacuation, and the occurrence of abdominal pain during defecation. All symptoms were rated as integers from 0 to 4, whereby higher scores represent greater severity of constipation, and the patients' self‐reported scores were aggregated by the researchers.

Data were collected from the patients through on‐site face‐to‐face interviews, telephone calls, or home visits by the research physicians. At each collection, any other adverse events or relevant laboratory values were recorded, mainly including nausea, vomiting, mental disorders, and hepatorenal function. When patients appeared to have serious or intolerable adverse events because of opioids, immediate symptom assessment was performed, and researchers advised patients to recheck with the oncology or pain department. If the attending physician made a relatively large change in a patient's opioid dosage or analgesic strategy, the patient was removed from our trial.

### 2.4 Outcomes

Our primary outcome was the increases in oral morphine equivalent daily doses (OMEDDs) between the two groups; the daily doses of every eligible participants were recorded at baseline and on days 7, 14, 21, 30, 60, and 90 of this trial, and the dosage changes were calculated on this basis. Secondary outcomes included dynamic changes in BTcP and average VAS pain scores, relief of anxiety and depression symptoms, remission of OIC (determined by comparing Wexner constipation scores), and the acceptability and safety of the L‐TAMS regimen.

### 2.5 Statistical analysis

Given the average onset time of oral L‐TAMS in a clinical trial by Liu et al.^9^ and an estimate^17^ of oral opioid dose changes based on our past clinical experiences treating patients with advanced cancer, we assumed that the baseline oral dose of morphine in the L‐TAMS and placebo groups would be 80 mg with a standard deviation of 20 and that the difference in dose change between the two groups would be 20% of the initial dose. With a two‐sided, two‐sample *t*‐test, 34 participants per group would be required to achieve 90% power to detect a statistically significant difference between the two groups. Assuming a 20% dropout rate, the final sample size necessary for each group was 43 participants.

The demographics and other general characteristics of the participants are presented in tabular form below. For continuous variables, the results are presented as the mean ± standard deviation (mean ± SD). Categorical variables are shown as the frequency/percentage (n/%). If the distribution was normal, the baseline characteristics of all measurement data were statistically analyzed by the t‐test of two independent samples; if not, the Mann–Whitney *U* test was applied. The chi‐square test or Fisher's exact test was employed to compare dichotomous data between two groups. Considering the interaction between intervention factors and duration of action, 2‐factor repeated‐measures analysis of variance was used to address increased daily oral morphine equivalents, VAS scores, PHQ‐9, GAD‐7 scores, and Wexner scores in each time. The Kruskal–Wallis *H* test was applied to compare the changes in BTcP over time between the two groups. In the case of inconsistent baselines of some population characteristics that may occur in the process of random computer allocation, covariance analysis would be used to handle some variables that may affect the statistical results to minimize the statistical bias caused by discrepant baselines. The Kolmogorov–Smirnov test was used to evaluate the normality of the data. The confidence limit was considered as 95%, and the statistical significance level was set as *p* < 0.05. Results were evaluated in the intention‐to‐treat (ITT) population and all participants under treatment were contained in the full analysis set (FAS). The data analysis was performed using SPSS (version 21, IBM Corporation.), and GraphPad Prism (version 7.0, GraphPad Software Corporation.) was applied in the production of statistical charts.

## 3 RESULTS

**FIGURE 1:**
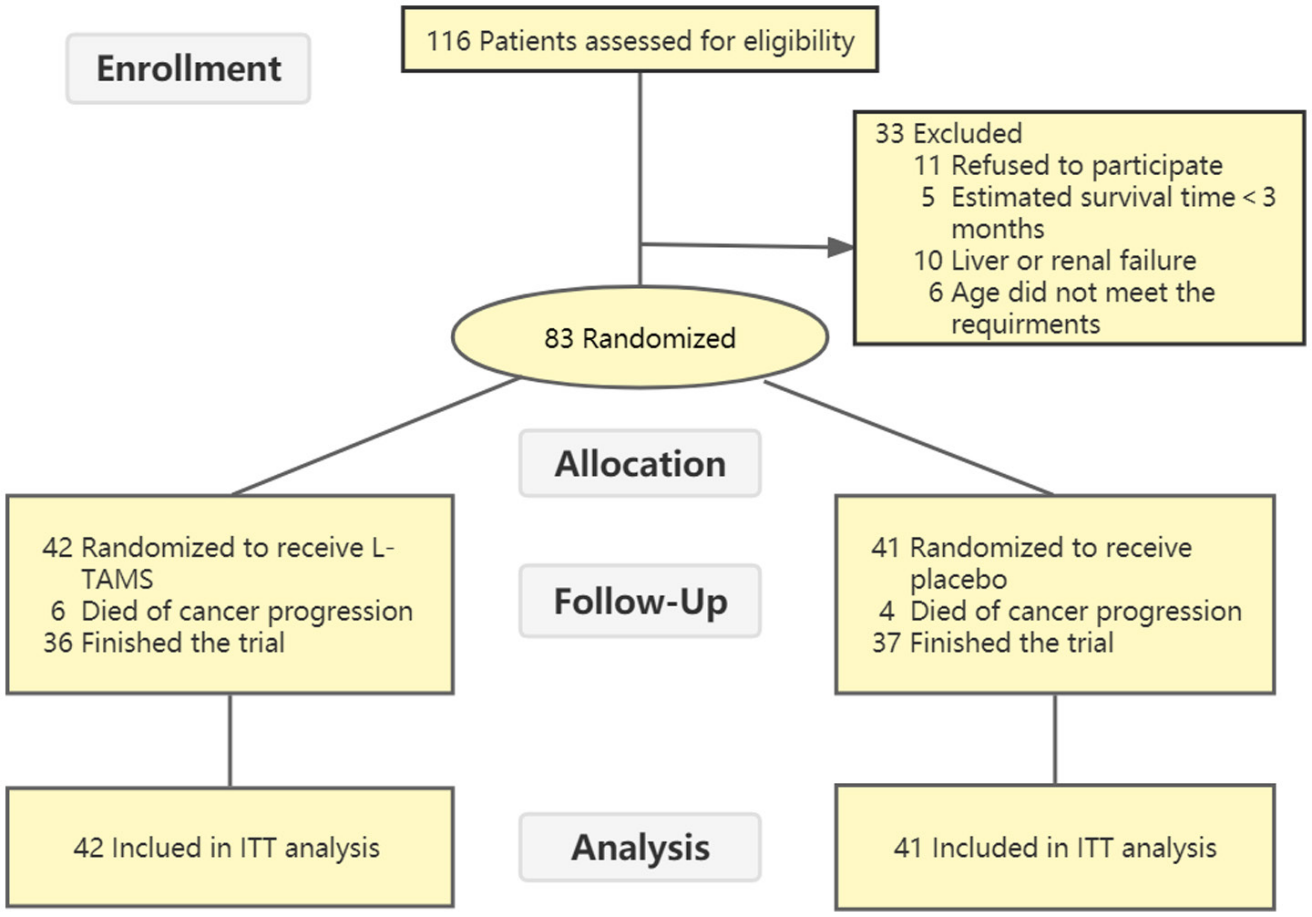
CONSORT flow diagram of the study participants. ITT, intention‐to‐treat

Between August 5, 2019 and June 30, 2020, a total of 116 patients from the oncology and pain departments of Xinhua Hospital Affiliated to Shanghai Jiao Tong University Medical College were screened, of whom 32 refused to sign informed consent or met at least one exclusion criterion for this trial. Finally, 83 patients with solid tumors who met the conditions were randomized into groups (No.: L‐TAMS group 42 vs. Placebo group 41). All patients completed the final follow‐up of this study. No data loss occurred during this trial except for 10 patients who died due to tumor progression (No.: L‐TAMS Group 6 vs. Placebo Group 4), whose data were also included in the final outcome analysis according to ITT principles (Figure 1). Except for Wexner score, all baseline characteristics of the participants were similar. (details can be seen in Table 1 and Table S1).

**TABLE 1:** Baseline characteristics of the patients

Characteristic	L‐TAMS (*n* = 42)	Placebo (*n* = 41)	*p* value
Age (SD), y	66.17 (12.66)	69.51 (11.36)	0.209
Male, No. (%)	24 (57.1%)	22 (53.7%)	0.750
BMI (SD), kg/m^2^	21.01 (4.08)	20.45 (3.26)	0.493
Primary solid tumor features			
Time since diagnosis (SD), months	24.17 (23.61)	24.32 (14.00)	0.972
History of Rad or Che, No. (%)	27 (64.3%)	21 (51.2%)	0.228
Osseous metastasis, No. (%)	17 (40.5%)	17 (41.5%)	0.927
Histogenesis			
Digestive system, No. (%)	15 (35.7%)	15 (36.7%)	0.934
Respiratory tract, No. (%)	13 (31.0%)	10 (24.4%)	0.504
Urogenital system, No. (%)	11 (26.2%)	15 (36.6%)	0.307
Others, No. (%)	3 (7.1%)	1 (2.4%)	0.306
Previous 24‐h total OMEDD (SD), mg/d	85.44 (44.47)	74.63 (33.85)	0.217
Baseline characteristics of cancer pain			
Mean VAS score in the past 24 h (SD)^a^	6.21 (1.44)	5.93 (0.93)	0.283
Mean BTcP in the past 24 h (SD)	2.33 (2.52)	1.51 (1.80)	0.093
Psychological assessment scores at enrolment			
PHQ‐9 (SD)^b^	15.29 (5.57)	15.17 (4.75)	0.920
GAD‐7 (SD)^c^	11.45 (4.49)	11.17 (3.40)	0.749
Assessment of OIC at enrolment			
Wexner score (SD)	20.14 (4.11)	17.37 (4.28)	0.003

Abbreviations: BMI, body mass index; BTcP, breakthrough cancer pain; Che, chemotherapy; GAD‐7, generalized anxiety disorder‐7; OMEDD, oral morphine equivalent daily dose; PHQ‐9, patient health questionnaire‐9; Rad, radiotherapy; VAS, visual analogue scale.

^a^ VAS:0–10, higher scores reveal more intense pain.

^b^ PHQ‐9:0–27, higher scores reveal more severe depressive symptoms.

^c^ GAD‐7:0–21, higher scores reveal more severe anxiety symptoms.

### 3.1 Primary outcomes

**FIGURE 2:**
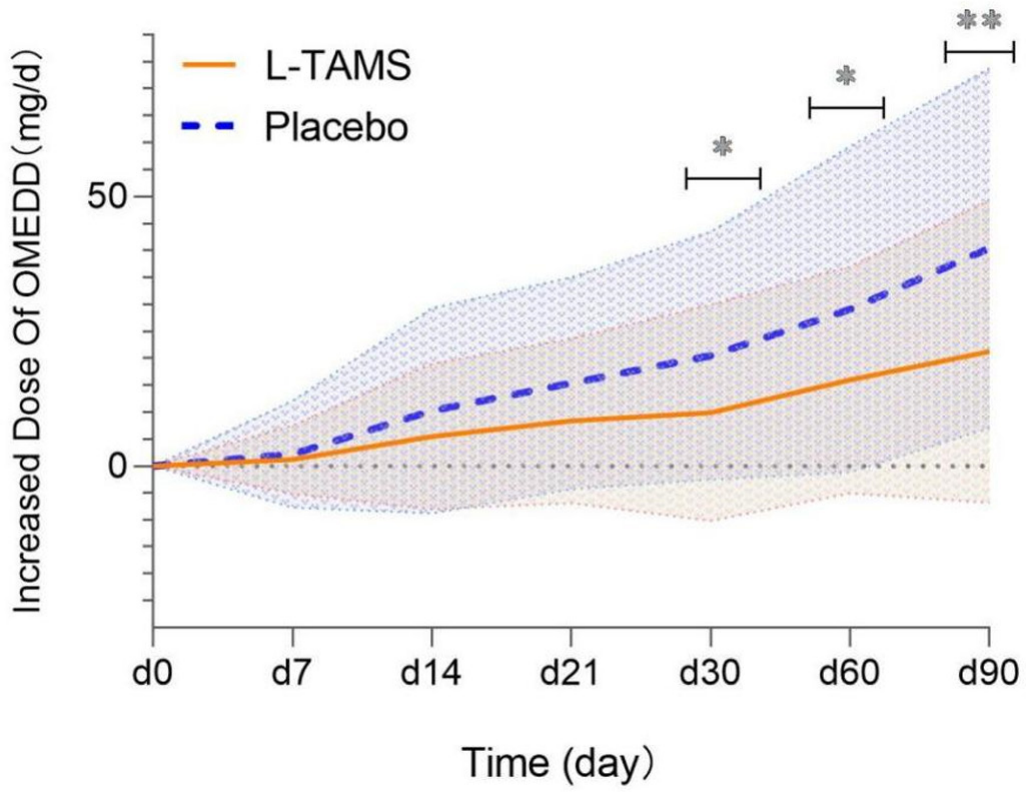
Comparison of OMEDD increases over time between the L‐TAMS group and the placebo group, shown as the mean ± SD (*: *p* < 0.05; **: *p* < 0.01). OMEDD, oral morphine equivalent daily dose

The two groups had similar baseline oral morphine equivalent daily doses (OMEDDs) (Table 1). The majority of patients in both groups did not experience changes in OMEDD during the first week of our trial, with the exception of individual participants who increased their doses (No.: L‐TAMS Group 3 vs. Placebo Group 5). On day 14, the OMEDDs in both groups increased significantly from baseline (L‐TAMS group: 90.92 ± 42.78 mg/d vs. 85.44 ± 44.48 mg/d, *p* < 0.05; Placebo group: 84.88 ± 44.39 mg/d vs. 74.63 ± 33.85 mg/d, *p* < 0.05), the average increase in OMEDD in the placebo group was greater than that in the L‐TAMS group, but no significant difference was found (L‐TAMS group 5.48 mg/d vs. Placebo group 10.24 mg/d, *p* = 0.19). The increase in OMEDD in the L‐TAMS group was significantly lower than that in the placebo group on day 30 (L‐TAMS group 9.85 mg/d vs. Placebo group 20.49 mg/d, *p* < 0.05), day 60 (L‐TAMS group 15.96 mg/d vs. Placebo group 29.06 mg/d, *p* < 0.05), and day 90 (L‐TAMS group 21.20 mg/d vs. Placebo group 40.44 mg/d, *p* < 0.01) (Figure 2 and Table S2).

### 3.2 Secondary outcomes

**FIGURE 3:**
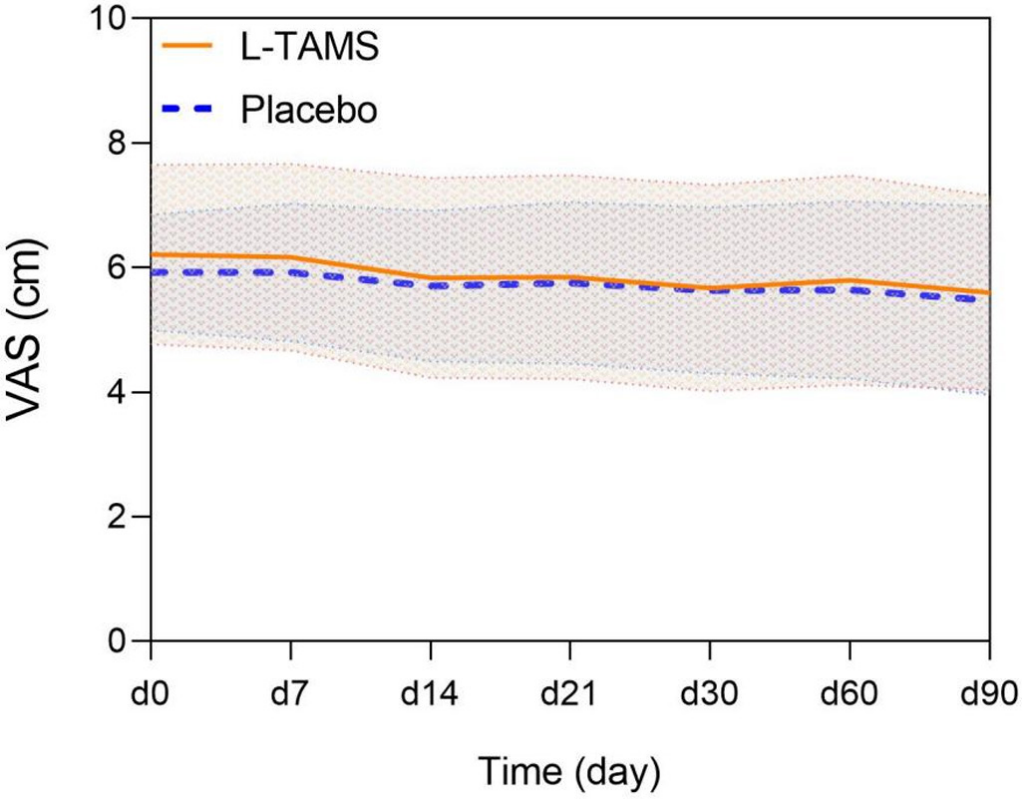
Comparison of mean daily VAS pain intensity scores between the L‐TAMS and placebo groups, shown as the mean ± SD. No obvious difference of VAS scores between the two groups was observed at any time point.VAS, visual analogue scale

The baseline VAS scores of the two groups were similar, and there was no significant difference in VAS scores between the two groups at any time after the beginning. However, VAS scores in both groups showed a decreasing trend compared with baseline, which in the L‐TAMS group were significantly lower than the baseline level since day 14 (L‐TAMS group 5.83 vs. Placebo group 6.21, *p* < 0.05), and in the placebo group showed statistically significant differences from baseline on day 90 (L‐TAMS group 5.47 vs. Placebo group 5.93, *p* < 0.05) (Figure 3 and Table S3);In terms of the BTcP, baseline values were similar between the two groups, with no significant differences at any time (7, 14, 21, 30, 60, or 90 days) after the start of this trial (Table S4).

**FIGURE 4:**
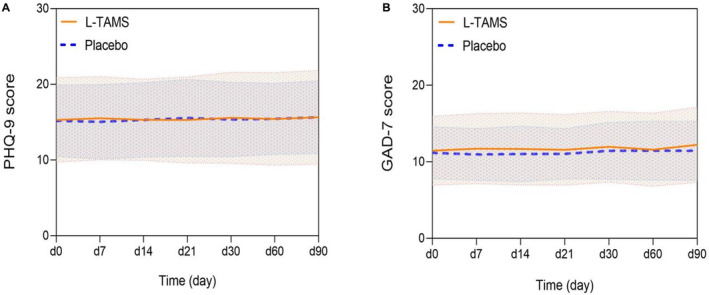
There was no significant difference in mood disorder symptoms (depression (A) or anxiety (B)) between the L‐TAMS and placebo groups; values are shown as the mean ± SD. PHQ‐9, patient health questionnaire‐9; GAD‐7, generalized anxiety disorder‐7

There was no significant difference in PHQ‐9 scores at any time point between the two groups and changes in PHQ‐9 scores compared with their respective baseline levels (Figure 4A and Table S5). There was also no significant difference in GAD‐7 scores between the two groups at any time. The average GAD‐7 scores on day 90 in the L‐TAMS group increased from baseline (12.22 vs. 11.45, *p* < 0.05), while the average GAD‐7 scores at any time in the placebo group did not change significantly (Figure 4B and Table S6).

**FIGURE 5:**
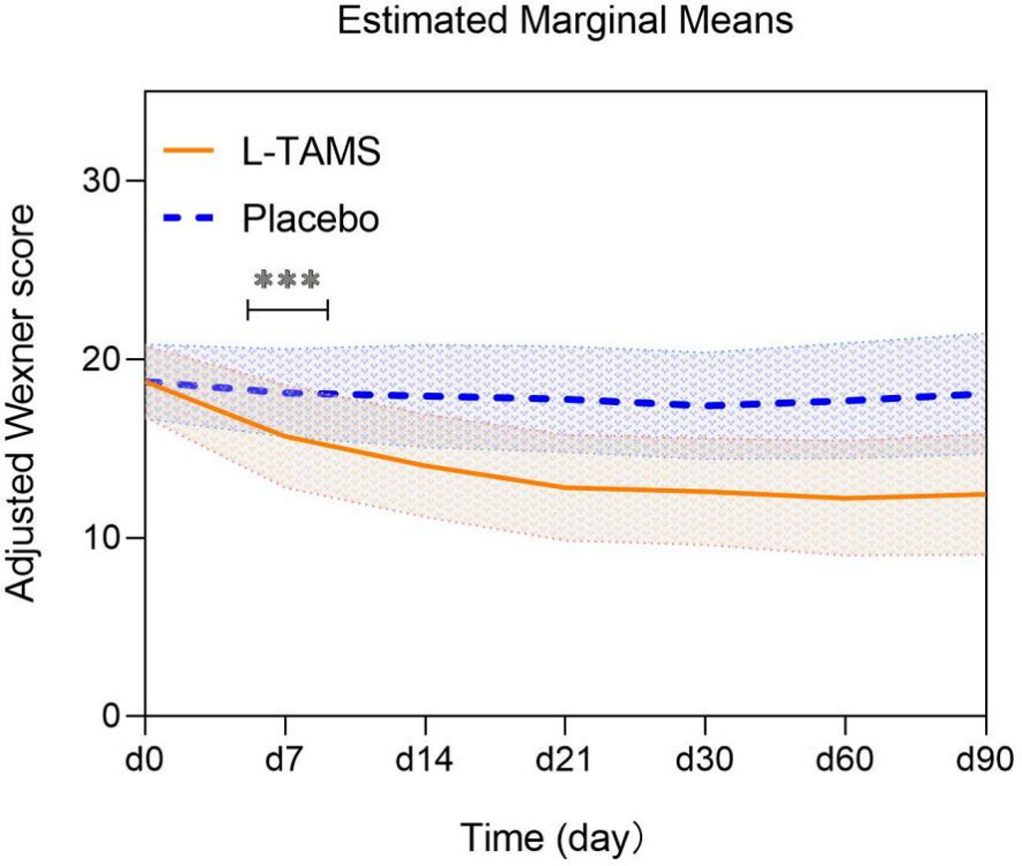
Comparison of adjusted Wexner constipation scores between the L‐TAMS and placebo groups after correction of baseline values by analysis of covariance. From day 7 to day 90, there was a significant score difference between the two groups. Values are shown as the mean ± SD (***: *p* < 0.001)

Considering the difference in the mean baseline Wexner scores between the two groups (L‐TAMS group 20.14 vs. Placebo group 17.37, *p* < 0.05), we applied the statistical method of covariance analysis to use the baseline Wexner score as the covariable of variance analysis. The results are presented as the mean ± SD to eliminate the interference of discrepant baselines with the statistical results as much as possible (initial values can be found in Table S7). The adjusted baseline Wexner constipation score of the two groups was 18.77. On day 7, the adjusted Wexner constipation score began to decrease significantly in the L‐TAMS group and barely changed in the placebo group, resulting in a statistically significant difference between the two groups (L‐TAMS group 15.70 vs. Placebo group 18.12, *p* < 0.001). The adjusted Wexner constipation score in the L‐TAMS group stabilized at 21 days after taking the medicine (L‐TAMS group 12.81 vs. Placebo group 17.78, *p* < 0.001). Compared with the L‐TAMS group, the adjusted Wexner constipation scores in the placebo group did not change significantly from baseline during the entire administration (Figure 5 and Table S8).

### 3.3 Adverse events

During our trial, no serious side effects, such as respiratory depression, sensorimotor disorder or heart block, occurred in the two groups. Aside from OIC, the most common side effect was nausea (L‐TAMS Group 8 vs. Placebo Group 10, *p* = 0.56, chi‐square test). Other common side effects included vomiting, pruritus, dizziness, and urine retention. There was no significant difference between the two groups in side effects (Table S9).

## 4 DISCUSSION

As far as we know, this is the first randomized controlled clinical trial to study the effects of an oral magnesium compound on opioid analgesia in patients with cancer pain. A systematic review has summarized the intravenous use of magnesium compounds (mainly MgSO_4_, 30 mg/kg–50 mg/kg) in the past 20 years on opioid analgesic effects (mainly for perioperative analgesia).^8^ The results show that magnesium can reduce total perioperative and postoperative pain and opioid consumption, with no serious adverse effects. However, the medication time of these studies is relatively short, and long‐term intravenous usage of MgSO_4_ may lead to nausea, vomiting, and heart block, especially for patients with advanced cancer who are often complicated with organ dysfunction.^1^, ^5^, ^18^ Based on this, repeated checks of neuromuscular reflexes and measurement of the blood concentration of magnesium are essential, which might limit its application in the clinic.^8^, ^19^, ^20^

Our results revealed that although the opioid dosage of the two groups continued to increase, a safe daily oral dose of L‐TAMS (1.5 g or 2.0 g/day) significantly reduced the increase in opioids in patients with advanced cancer compared with the placebo group starting on day 30. On day 90, this difference was even more pronounced. This is consistent with the conclusion that magnesium can reduce the dosage of opioids and ease the development of opioid tolerance.^6^, ^7^, ^8^, ^18^ L‐TAMS differs markedly from other common magnesium‐based drugs in that it is taken orally rather than injected intravenously. Oral L‐TAMS is a safe drug with a high absorption rate and retention rate in vivo, improving the concentration of free magnesium in cerebrospinal fluid and neurons through the blood–brain barrier.^9^, ^10^, ^21^, ^22^ However, given the slow uptake and susceptibility to the gastrointestinal function of oral administration, this approach took significantly longer to act than intravenous administration.^9^, ^22^

The mechanism of the effect of Mg^2+^ on opioid tolerance is not very clear. Conventional wisdom holds that Mg^2+^ is a natural antagonist of the N‐methyl‐D‐aspartic acid receptor (NMDAR), can physically block the Ca^2+^ channels coupled with NMDARs, and inhibits Ca^2+^/CaMKII‐dependent nitric oxide synthase (NOS) activity.^23^, ^24^, ^25^ Decreased NO production ultimately affects intracellular cGMP production, which, in turn, is manifested as enhanced morphine analgesia through changes in cGMP‐dependent protein kinase activity.^7^, ^24^ Moreover, the activation of NMDAR can also cause the activation of protein kinase C (PKC), which plays a vital role in the development of morphine tolerance. The activity of cPKC is also affected by intracellular Ca^2+^ and DG concentrations, and there are positive regulatory sites for cPKC on NMDARs, resulting in positive feedback.^26^ In addition to the mechanism based on NMDARs, a recent molecular simulation experiment confirmed that in the presence of a physiological concentration of Na^+^, a slightly increased concentration of extracellular Mg^2+^ could preferentially bind to the extracellular part of the μ‐opioid receptor and regulate the G‐protein‐binding region of the receptor to promote the maintenance of receptor activity. Thus, the dissociation effect of agonists was weakened, which enhanced the binding affinity of agonists and the analgesic effect of opioids.^27^

Moreover, it was observed in our study that constipation in the L‐TAMS group was significantly relieved compared to that in the placebo group on day 7. The adjusted Wexner constipation score in the L‐TAMS group continued to decrease until day 21, while the Wexner score in the placebo group was not significantly improved. In addition to the classic osmotic cathartic effect of magnesium salts,^28^ the oral absorption of L‐TAMS also provides other possible explanations. Ikarashi et al. confirmed that an increase in the expression level of Aquaporin 3 (AQP 3) is involved in the onset of morphine‐induced constipation, while an increased intracellular Mg^2+^ concentration can weaken this effect and promote the water inflow of intestinal mucosal epithelial cells into the intestinal cavity, which is conducive to defecation.^29^ Since we did not set the efficacy comparison between L‐TAMS and other classical laxatives, it is not reasonable to directly determine whether L‐TAMS has a better effect on constipation, which may need further research.

There were no significant differences in VAS score or the frequency of BTcP between the two groups, but it is interesting to note that the VAS scores of the two groups all declined from baseline, and we assume that some of the patients did not have enough pain relief before this trial began. Although L‐TAMS can help reduce morphine dosage and ease the development of opioid tolerance, prescribed opioid dosage often fails to meet the analgesic needs of some advanced cancer patients orally. There were no significant differences in PHQ‐9 and GAD‐7 scores between the two groups, suggesting that oral L‐TAMS cannot relieve mental disorders in patients with advanced cancer. We did not find statistically significant differences between the two groups in other adverse events except for constipation. On the whole, this dosage of L‐TAMS is safe. A systematic review of studies on oral magnesium preparation for the treatment of hypertension showed that no severe adverse reactions were observed after oral doses equivalent to as much as 972 mg magnesium per day, which was much higher than our trial dosage, and most reported adverse effects were minor and transient.^30^

Our trial had some limitations. First, because of the complexity of participants' pain and the lack of similar references, there were no exact criteria for dose escalation in this trial, which needs to be improved in subsequent studies. Second, our results suggested that the effect of L‐TAMS on morphine tolerance may be slow, but at the same time, paradoxically, patients with advanced cancer do not allow us to set excessively long data collection timelines. Third, we did not measure patients' blood magnesium levels or their changes. However, a long period of clinical experience has shown that serum magnesium concentration does not reflect its concentration in other tissues in a repeatable and credible method,^31^, ^32^ and more effective tests may be needed in the future to clarify the dose‐effect relationship. Last but not least, our sample size was small, and due to the withdrawal of several patients, the target sample capacity was not reached, which limited the interpretation of our results to some extent.

In conclusion, our study shows that safe oral dosage of L‐TAMS can improve the analgesic effect of opioids and reduce the dosage of opioids used in patients with advanced cancer while significantly relieving OIC without significant toxicity or side effects. More clinical samples and longer follow‐up period are necessary to determine the impact of oral L‐TAMS on opioid tolerance in cancer patients.

## AUTHOR CONTRIBUTIONS

Design and development of the protocol: Siyin Wu, Tian Jin, Boris V. Krylov, Xianguo Liu, Ke Ma. Acquisition of data:Siyin Wu, Tian Jin,Bingjie Ma, Yun Ji. Data analysis and visualization:Siyin Wu,Xuehua Huang, Peiliang Wang, Xiaoming Liu, Boris V. Krylov, Xianguo Liu, Ke Ma. Writing, review and revision of the manuscript:All authors. Study supervision:Siyin Wu, Tian Jin, Bingjie Ma, Yun Ji, Xuehua Huang, Xiaoming Liu, Boris V. Krylov, Xianguo Liu, Ke Ma. Project administration:Xuehua Huang, Peiliang Wang, Xiaoming Liu, Boris V. Krylov, Xianguo Liu, Ke Ma.

## FUNDING INFORMATION

No specific funding was disclosed.

## CONFLICT OF INTEREST

The authors declared no potential conflicts of interest.

## ETHICAL APPROVAL STATEMENT AND CLINICAL TRIAL REGISTRATION NUMBER

Ethical approval for the trial was obtained from the Medical Ethics Committee of Xinhua Hospital Affiliated with Shanghai Jiao Tong University Medical College, and the implementation of this trial was in accordance with the Quality Management Standards for Drug Clinical Trials, the Declaration of Helsinki and relevant Chinese laws and regulations. All patients provided written informed consent before enrolment. This trial was registered in the International Standard Randomized Controlled Trial Number (ISRCTN, www.isrctn.com) registry (No. ISRCTN60931429).

## Supporting information


Appendix S1

Appendix S2

## Data Availability

The data generated in this study are available upon request from the corresponding author.
