# Supplementary material for: Oral application of magnesium‐L‐threonate enhances analgesia and reduces the dosage of opioids needed in advanced cancer patients—A randomized, double‐blind, placebo‐controlled trial
Source: Cancer Med. 2023 Jan 26;12(4):4343–51. doi: 10.1002/cam4.4922 (PMC9972038; doi:10.1002/cam4.4922)
Supplement: Supplementary file 1 — Appendix S1 [file CAM4-12-4343-s001.docx]

Inclusion criteria: patients must meet all the following conditions：

1) Patients with cancer pain, taking morphine sulfate sustained-release tablets or other opioids orally (oral dose: 20-200 mg morphine equivalents/24 h), could signed the informed consent for this trial voluntarily;

2) Patients were 18 – 80 years old, regardless of men and women, VAS> 4 points of cancer pain, or BTcP> 3 times / day;

3) The survival time was expected to exceed 3 months

4) Patients could follow the drug dosage and visit plan;

5) Could objectively describe the symptoms, had no serious infection, respiratory insufficiency, etc., and could actively cooperate;

6) No allergic diseases, non-allergic constitution;

7) Patients had no contraindications for taking L-TAMS;

8) Patients without drug abuse or drug addiction;

9) Did not participate in drug trials within 3 months before this trial (including this trial drug)

Exclusion criteria:

1) Those who failed to meet the inclusion standards;

2) Patients with severe respiratory system, cardiovascular system diseases, liver and renal dysfunction;

3) Patients had significant traumatic pain or postoperative incision pain in addition to cancer pain.

4) Breast-feeding and pregnant women, patients with pregnancy plans within 1 month after the trial (also including the male patients);

5) Patients who have participated in the drug test within 3 months before the trial (including the trial drugs);

6) Sponsors or researchers or their family members directly involved in this trial;

7) The researchers believed that some patients could not be selected for other reasons.
