# Supplementary material for: Oral application of magnesium‐L‐threonate enhances analgesia and reduces the dosage of opioids needed in advanced cancer patients—A randomized, double‐blind, placebo‐controlled trial
Source: Cancer Med. 2023 Jan 26;12(4):4343–51. doi: 10.1002/cam4.4922 (PMC9972038; doi:10.1002/cam4.4922)
Supplement: Supplementary file 2 — Appendix S2 [file CAM4-12-4343-s002.docx]

| **Table 1. Baseline Characteristics of the Patients.** | | | |
| --- | --- | --- | --- |
| Characteristic | L-TAMS (n=42) | Placebo (n=41) | P value |
| Age (SD), y | 66.17(12.66) | 69.51(11.36) | 0.209 |
| Male, No. (%) | 24(57.1%) | 22(53.7%) | 0.750 |
| BMI (SD), kg/m^2^ | 21.01(4.08) | 20.45(3.26) | 0.493 |
| Primary solid tumour features | | | |
| Time since diagnosis (SD), months | 24.17(23.61) | 24.32(14.00) | 0.972 |
| History of Rad or Che, No. (%) | 27(64.3%) | 21(51.2%) | 0.228 |
| Osseous metastasis, No. (%) | 17(40.5%) | 17(41.5%) | 0.927 |
| Histogenesis | | | |
| Digestive system, No. (%) | 15(35.7%) | 15(36.7%) | 0.934 |
| Respiratory tract, No. (%) | 13(31.0%) | 10(24.4%) | 0.504 |
| Urogenital system, No. (%) | 11(26.2%) | 15(36.6%) | 0.307 |
| Others, No. (%) | 3(7.1%) | 1(2.4%) | 0.306 |
| Previous 24-h total OMEDD (SD), mg/d | 85.44(44.47) | 74.63(33.85) | 0.217 |
| Baseline characteristics of cancer pain |  |  |  |
| Mean VAS score in the past 24 h (SD)^a^ | 6.21(1.44) | 5.93(0.93) | 0.283 |
| Mean BTcP in the past 24 h (SD) | 2.33(2.52) | 1.51(1.80) | 0.093 |
| Psychological assessment scores at enrolment | | | |
| PHQ-9 (SD)^b^ | 15.29(5.57) | 15.17(4.75) | 0.920 |
| GAD-7 (SD)^c^ | 11.45(4.49) | 11.17(3.40) | 0.749 |
| Assessment of OIC at enrolment |  |  |  |
| Wexner score (SD) | 20.14(4.11) | 17.37(4.28) | 0.003 |

Abbreviations: BMI, body mass index; Rad, radiotherapy; Che, chemotherapy; OMEDD, oral morphine equivalent daily dose; VAS, visual analogue scale; BTcP, breakthrough cancer pain; PHQ-9, Patient Health Questionnaire-9; GAD-7, Generalized Anxiety Disorder-7.

^a^VAS scores can range from 0 to 10, with higher scores indicating more intense pain.

^b^PHQ-9 scores can range from 0 to 27, with higher scores indicating more severe depressive symptoms.

^c^GAD-7 scores can range from 0 to 21, with higher scores indicating more severe anxiety symptoms.

| Table 2.Comparison of OMEDD increments between groups at 7, 14, 21, 30, 60 and 90 days after treatment,mg/d .(Mean±SD) | | | |
| --- | --- | --- | --- |
| Time L-TAMS(n=42) Placebo(n=41) P Value | | | |
| 7 d | 1.19±6.33 | 2.20±9.88 | 0.582 |
| 14 d | 5.48±13.47 | 10.24±19.04 | 0.191 |
| 21 d | 8.39±15.30 | 15.37±19.63 | 0.074 |
| 30 d | 9.85±20.07 | 20.49±23.02 | 0.027 |
| 60 d | 15.96±21.03 | 29.06±30.23 | 0.024 |
| 90 d | 21.20±28.05 | 40.44±33.36 | 0.006 |

| Table 3.Comparison of VAS score between groups at baseline ,7, 14, 21, 30, 60 and 90 days after treatment (Mean±SD). | | | |
| --- | --- | --- | --- |
| time | L-TAMS | Placebo | P |
| baseline | 6.21±1.44 | 5.93±0.93 | 0.285 |
| d7 | 6.17±1.50 | 5.93±1.10 | 0.409 |
| d14 | 5.83±1.61 | 5.71±1.21 | 0.688 |
| d21 | 5.85±1.63 | 5.76±1.30 | 0.779 |
| d30 | 5.67±1.65 | 5.63±1.34 | 0.906 |
| d60 | 5.80±1.68 | 5.64±1.43 | 0.639 |
| d90 | 5.60±1.56 | 5.47±1.51 | 0.712 |

| Table 4.Comparison of BTcP between groups at baseline ,7, 14, 21, 30, 60 and 90 days after treatment (NO.)(Mean±SD). | | | |
| --- | --- | --- | --- |
| time | L-TAMS | Placebo | P |
| baseline | 2.33±2.52 | 1.51±1.80 | 0.093 |
| d7 | 2.07±2.52 | 1.51±1.98 | 0.265 |
| d14 | 2.02±2.40 | 1.46±1.90 | 0.243 |
| d21 | 1.97±2.22 | 1.49±1.85 | 0.285 |
| d30 | 2.02±2.41 | 1.46±1.96 | 0.250 |
| d60 | 2.11±2.33 | 1.46±1.94 | 0.177 |
| d90 | 2.06±2.28 | 1.41±1.98 | 0.166 |

| Table 5.Comparison of PHQ-9 score between groups at baseline ,7, 14, 21, 30, 60 and 90 days after treatment (Mean±SD). | | | |
| --- | --- | --- | --- |
| time | L-TAMS | Placebo | P |
| baseline | 15.29±5.57 | 15.17±4.75 | 0.920 |
| d7 | 15.52±5.50 | 15.05±4.93 | 0.680 |
| d14 | 15.31±5.35 | 15.29±4.93 | 0.988 |
| d21 | 15.28±5.69 | 15.56±5.10 | 0.816 |
| d30 | 15.57±6.02 | 15.34±4.94 | 0.853 |
| d60 | 15.41±6.11 | 15.44±4.71 | 0.981 |
| d90 | 15.64±6.20 | 15.66±4.82 | 0.991 |

| Table 6. Comparison of GAD-7 score between groups at baseline ,7, 14, 21, 30, 60 and 90 days after treatment (Mean±SD). | | | |
| --- | --- | --- | --- |
| time | L-TAMS | Placebo | P |
| baseline | 11.45±4.49 | 11.17±3.40 | 0.749 |
| d7 | 11.71±4.60 | 10.98±3.39 | 0.408 |
| d14 | 11.69±4.70 | 11.02±3.62 | 0.473 |
| d21 | 11.56±4.63 | 11.05±3.29 | 0.564 |
| d30 | 11.96±4.64 | 11.44±3.72 | 0.577 |
| d60 | 11.59±4.78 | 11.46±3.87 | 0.898 |
| d90 | 12.22±4.92 | 11.44±3.85 | 0.424 |

| Table 7. Comparison of initial Wexner score for OIC between two groups at baseline, 7, 14, 21,30, 60 and 90 days after treatment. | | |
| --- | --- | --- |
| time | L-TAMS | Placebo |
| baseline | 20.14 | 17.37 |
| d7 | 16.83 | 16.95 |
| d14 | 15.00 | 16.98 |
| d21 | 13.57 | 17.00 |
| d30 | 13.22 | 16.78 |
| d60 | 12.86 | 17.05 |
| d90 | 13.09 | 17.43 |

| Table 8. Comparison of adjusted Wexner score for OIC between groups at 7, 14, 21, 30, 60 and 90 days after treatment .(Mean±SD) | | | |
| --- | --- | --- | --- |
| Time L-TAMS(n=42) Placebo(n=41) P Value | | | |
| baseline 18.77±2.08 18.77±2.12 | | | |
| 7 d | 15.70±2.46 | 18.12±2.47 | ＜0.001 |
| 14 d | 14.04±2.87 | 17.96±2.88 | ＜0.001 |
| 21 d | 12.81±2.97 | 17.78±2.97 | ＜0.001 |
| 30 d | 12.60±2.99 | 17.41±3.00 | ＜0.001 |
| 60 d | 12.24±3.22 | 17.69±3.22 | ＜0.001 |
| 90 d | 12.45±3.37 | 18.09±3.37 | ＜0.001 |

| Table 9.Comparison of adverse events(AEs) between the two groups.(Chi-square test or Fisher Exact test) | | | |
| --- | --- | --- | --- |
| AEs L-TAMS(n=42) Placebo(n=41) P Value | | | |
| Nausea | 8(19.1%) | 10(24,4%) | 0.555 |
| Vomiting | 4(9.3%) | 5(12.2%) | 0.969 |
| Pruritus | 2(4.8%) | 3(7.3%) | 0.978 |
| Dizziness | 3(7.1%) | 2(4.9%) | 1.000 |
| Urine retention | 0(0%) | 1(2.4%) | 0.494 |
